# The effectiveness of isometric protocols using an external load or voluntary effort on jump height enhancement in trained females

**DOI:** 10.1038/s41598-023-40912-0

**Published:** 2023-08-19

**Authors:** Dawid Koźlenia, Jarosław Domaradzki

**Affiliations:** Unit of Biostructure, Faculty of Physical Education and Sport, Wroclaw University of Health and Sport Sciences, al. I.J. Paderewskiego 35, 51-612 Wrocław, Poland

**Keywords:** Physiology, Environmental sciences

## Abstract

This study aimed to examine the effectiveness of isometric post-activation performance enhancement protocols using an external load (EXL) or voluntary effort (VE) on jump height (JH) in trained females divided into EXL (n = 15), VE (n = 14), and control (CON; n = 12) groups. JH was assessed using countermovement jumps at baseline and the third, fifth, seventh, and ninth minutes after the protocols. The EXL performed three sets of back squats with a 70%-repetition maximum load for four seconds, with one-minute breaks. The VE performed three sets of pushing against an immovable bar in the back squat position for five seconds, with one-minute breaks. The CON group ran on a treadmill at 6 km/h for four minutes. A RM-ANOVA showed a significant interaction for group-time (*p <* 0.01). The EXL protocol provided JH improvement at the third minute compared to baseline (*p =* 0.01), though it decreased in subsequent minutes (*p <* 0.05). JH declined in the VE group at the third and fifth minutes (*p <* 0.05), then peaked, surpassing baseline, after nine minutes (*p =* 0.04). No significant differences were found between the protocols in the relative effect (best—baseline) (*p =* 0.09), though the EXL group appeared to gain more (effect size [ES] = 0.76). Both protocols improved JH, but caution is required due to peak performance time and potential JH reduction.

## Introduction

Jump height (JH) is one of the most reliable and informative assessments associated with muscle power, a crucial factor in sports performance^1,2^. Therefore, JH measurements are widely utilized in various sports disciplines for training process monitoring, with JH improvement also developed as an independent skill^1,3^. A study by Pedersen et al.^4^ showed a strong relationship between JH and physical match performance in high-level female football players. Meanwhile, Carlock et al.^5^ indicated that jumping ability corresponded with weightlifting results. Also, untrained males and females displayed a strong dependence between jump ability and physical performance^6^. Cormie et al.^7^ demonstrated improved jump performance after a few weeks of resistance training. However, improvement in jump performance can be achieved acutely, and not only through longitudinal training programmes^7,8^.

Post-activation performance enhancement (PAPE) protocols can acutely improve power in physical performance by increasing neural drive, elevating body temperature, and enhancing the flow of intracellular fluids^8–10^. Achieving these benefits requires consideration of factors such as load intensity and volume and an appropriate rest interval to avoid suppression of performance enhancement due to fatigue. Additionally, the effort used in the protocol should be related to the targeted activity^8–10^. It is suggested that short, high-intensity effort, with approximately three or more minutes of rest, should produce visible performance enhancement^8^. Using PAPE in training or competition may enhance physical performance^11,12^ and could be treated as the main target for maximizing performance during warm-up^10^.

The type of muscle contraction used in PAPE protocols, such as concentric, eccentric, and isometric, are under discussion. All can provide positive results, though no consensus exists on which type is superior^13,14^. Isometric contractions are relatively safe and, despite their static nature, may translate into dynamic action improvement^15^. Isometric muscle action is characterized by low energy expenditure^16^, although some ambiguity exists over isometric PAPE protocols^17^. Some authors have used a relative external load (EXL)^18^, whereas other protocols are based on voluntary effort (VE)^19^, which can include hold isometric muscle action (HIMA) and push isometric muscle action (PIMA), respectively^20^. VE is considered safer and is based more on individual ability and effort engagement^21–23^, though it requires more time to achieve an appropriate intensity, which may need prolonged stimulation^24^. In contrast, an EXL may bring about faster fatigue, meaning caution is needed with muscle activity duration^20^. Regardless, both approaches can provide positive effects, but no study has directly compared isometric muscle actions in performance enhancement.

The data on isometric PAPE protocols among females are limited, although most other contraction types show positive effects in both sexes^25–27^. Villalon-Gasch et al.^12^ observed increased JH among female volleyball players after a conditioning activity protocol based on dynamic activity. Spieszny et al.^19^ showed JH improvement in team sports players after isometric muscle action, with variability in peak performance, but the study only involved males. Rixon et al.^13^ demonstrated improvement in JH after voluntary isometric effort in males and females, but Tsolakis et al.^27^ did not observe any benefits after introducing the protocol with HIMA. Due to limited observations in females, it is difficult to refer to any isometric conditioning activity protocol with an EXL. However, positive effects were observed in males^18^.

The data comparing the effectiveness of an EXL and VE during isometric protocols is limited, and how females respond to these types of protocols remains unclear. The results of the current study could offer practitioners valuable insights into the effective use of isometric PAPE protocols in various settings—using either an EXL or VE. Indeed, the findings may provide practical guidelines for enhancing jump performance when preparing for training sessions or competitions. Due to the ambiguity in the studies outlined above and the gaps in the literature, this study aimed to establish the effectiveness of isometric PAPE protocols using and EXL or VE on JH enhancement in trained females. Specifically, we ask (1) if the introduced protocols effectively enhance JH. (2) Are there different effects of the protocols on absolute improvement (best—baseline)? (3) Is there a difference in time to peak performance? We hypothesized that both protocols could improve JH, but peak performance time would differ between the two. The results provided fill gaps in the literature, where there is a lack of observations directly comparing protocols based on various isometric muscle actions and limited data considering PAPE protocols’ effects on females.

## Results

Based on the results of the chi-squared analysis of the group number inequality, we can conclude that the observed difference in the number of participants in each group (15 vs. 14 vs. 12) were not significantly different (χ^2^ = 1.105, *p =* 0.58).

Table 1 provides a statistical description of the participants in each group and countermovement jump (CMJ) height changes (Δ) over the subsequent minutes. Comparisons of variables using one-factor (group) analysis of variance (ANOVA) found no significant differences between the groups (F = 1.5; eta-squared [η^2^] = 0.24; *p =* 0.13).

**Table 1:** Descriptive statistics of study participants according to group adherence and jump height changes (Δ) over subsequent minutes.

Variable	External load (EXL) n = 15	Voluntary effort (VE) n = 14	Control (CON) n = 12
	Mean ± SD (95%CI)	Mean ± SD (95%CI)	Mean ± SD (95%CI)
Age (years)	21.07 ± 1.83 (20.05–22.08)	20.71 ± 1.38 (19.92–21.51)	21.5 ± 0.67 (21.07–21.93)
Body height (m)	1.7 ± 0.07 (1.67–1.74)	1.67 ± 0.06 (1.64–1.71)	1.69 ± 0.04 (1.66–1.71)
Body mass (kg)	60.49 ± 7.09 (56.56–64.42)	60.94 ± 9.83 (55.27–66.62)	60.75 ± 6.21 (56.8–64.7)
Body mass index (kg/m^2^)	20.84 ± 1.76 (19.86–21.82)	21.82 ± 3.49 (19.8–23.83)	21.26 ± 2.33 (19.78–22.74)
Training experience (years)	4.2 ± 1.9 (3.15–5.25)	4 ± 1.24 (3.28–4.72)	4.71 ± 1.50 (3.22–5.23)
Weekly training volume [min/week]	126.33 ± 69.45 (87.87–164.79)	120.42 ± 51.72 (87.56–153.28)	132.14 ± 53.09 (101.49–162.79)
Back squat relative strength [1RMax-kg/body weight-kg]	1.14 ± 0.09 (1.09–1.19)	1.12 ± 0.13 (1.05–1.19)	1.18 ± 0.09 (1.12–1.24)

CI, confidence interval; RM, repetition maximum; SD, standard deviation.

Table 2 presents CMJ measures values in consecutive minutes for all three groups.

**Table 2:** Countermovement jump (CMJ) results according to group adherence measured in consecutive minutes.

Variable (cm)	EXL Mean ± SD (95%CI)	VE Mean ± SD (95%CI)	CON Mean ± SD (95%CI)
CMJ baseline	23.99 ± 3.99 (21.78–26.2)	24.52 ± 3.57 (22.46–26.58)	24.42 ± 3.22 (22.37–26.47)
CMJ 3′	25.13 ± 4.03 (22.90–27.36)	23.54 ± 3.06 (21.77–25.31)	23.86 ± 3.50 (21.64–26.08)
CMJ 5′	24.11 ± 3.78 (22.01–26.2)	23.79 ± 2.99 (22.06–25.52)	24.32 ± 3.47 (22.12–26.53)
CMJ 7′	24.01 ± 4.48 (21.53–26.5)	24.34 ± 3.11 (22.55–26.14)	23.82 ± 3.57 (21.55–26.09)
CMJ 9′	23.46 ± 4.45 (21.00–25.92)	25.24 ± 3.79 (23.06–27.43)	23.90 ± 3.65 (21.58–26.22)

**Figure 1:**
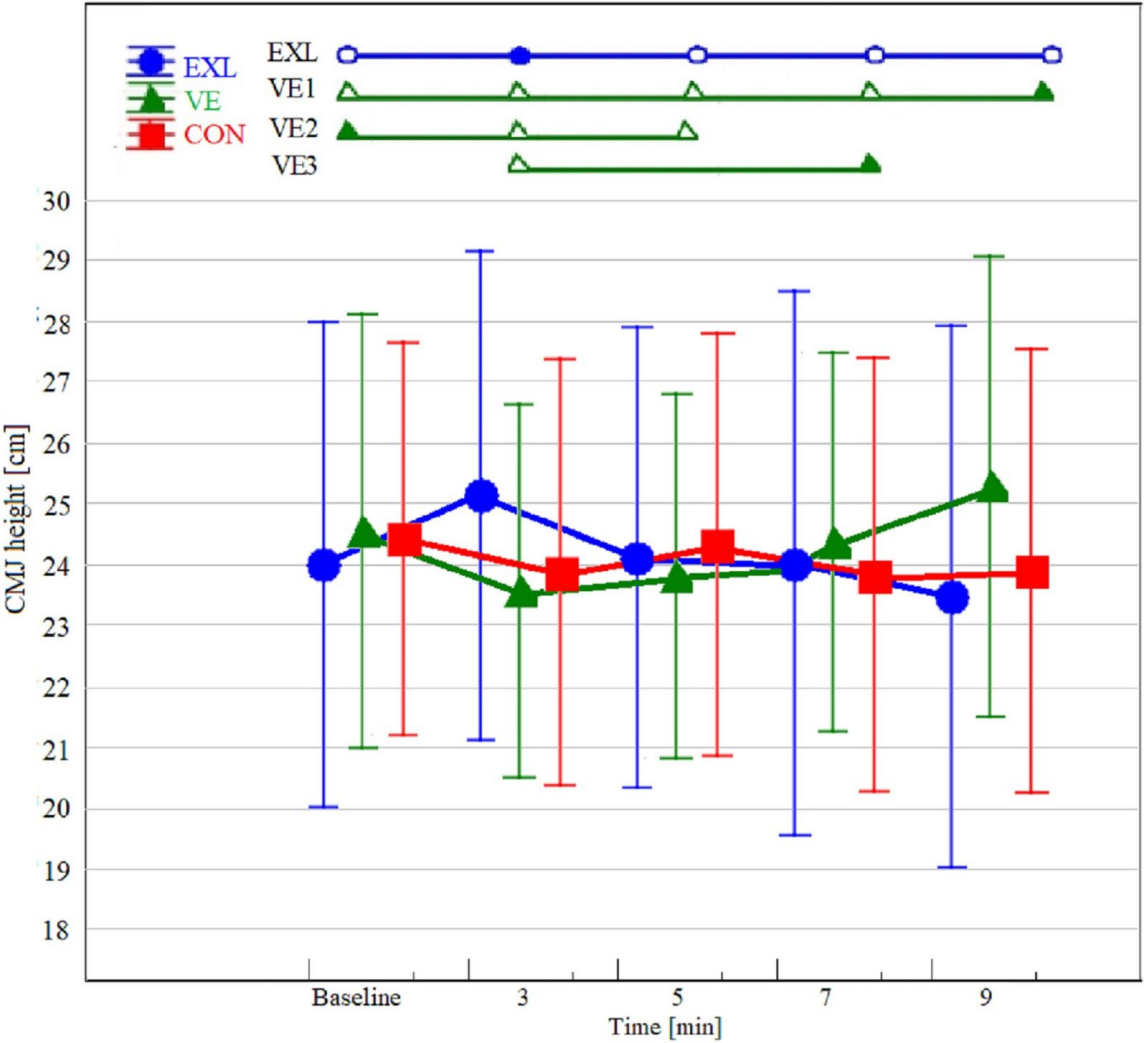
Jump height changes after PAPE intervention according to group adherence. Points represent means and vertical lines represent standard deviations. Metro plots indicate statistically significant differences (*p <* 0.05). The full point in the metro plot indicates statistically significantly higher result than empty one in the same line. EXL: 3rd > baseline *p =* 0.01; 3rd > 5th *p <* 0.01; 3rd > 7th *p =* 0.01; 3rd > 9th *p <* 0.01. VE1: 9th > baseline *p =* 0.04; 9th > 3rd *p <* 0.01; 9th > 5th *p <* 0.01; 9th > 7th *p =* 0.01. VE2: Baseline > 3rd *p <* 0.01; Baseline > 5th *p <* 0.39. VE3: 7th > 3rd *p =* 0.02.

The two-factor repeated measures ANOVA revealed a significant interaction between group and time (F = 7.1; η^2^ = 0.27; *p <* 0.01). Post hoc comparison indicated a marked improvement in jump performance in the EXL group in the third minute compared to baseline (*p =* 0.01), which was also the peak performance time. Indeed, performance in the third minute was significantly better than in the fifth (*p <* 0.01), seventh (*p =* 0.01), and ninth minutes (*p <* 0.01). The VE group performance peaked in the ninth minute, with a significantly higher jump than baseline (*p =* 0.04) and third (*p <* 0.01), fifth (*p <* 0.01), and seventh minutes (*p =* 0.01). In addition, JH performance in the third minute decreased compared to the baseline (*p <* 0.01) and seventh minute (*p =* 0.02). This deteriorative effect also occurred in the fifth minute, when the performance fell below baseline (*p =* 0.39). The CON group experienced only non-significant changes. Figure 1 presents the JH results from the baseline and in the minutes following post-activation with details of statistically significant differences.

The next step in the analysis considered if peak performance depended on time, with chi-squared analysis confirming (χ^2^ = 16.33; *p =* 0.04) that the EXL group peaked more frequently in the third (n = 5) and fifth minutes (n = 4), whereas the VE group needed until the ninth minute (n = 9). The best results were observed in the CON group at the baseline (n = 3) and ninth minute (n = 4).

**Figure 2:**
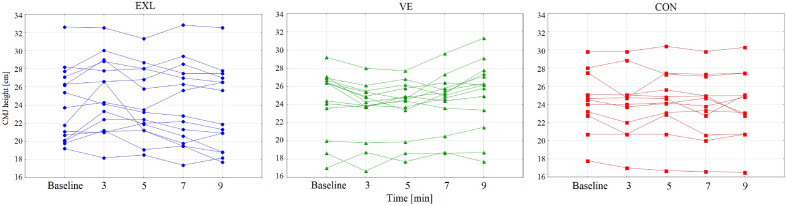
Individual responses on protocols.

The final analysis compared the absolute improvements between the EXL and VE protocols, using the difference (Δ) between the best results and baseline. Although both protocols are characterized by optimal moment-to-peak performance, not all individuals peaked simultaneously, showing a high variation coefficient for the time-to-peak performance of 60% for EXL and 57.5% for VE (Fig. 2).

**Figure 3:**
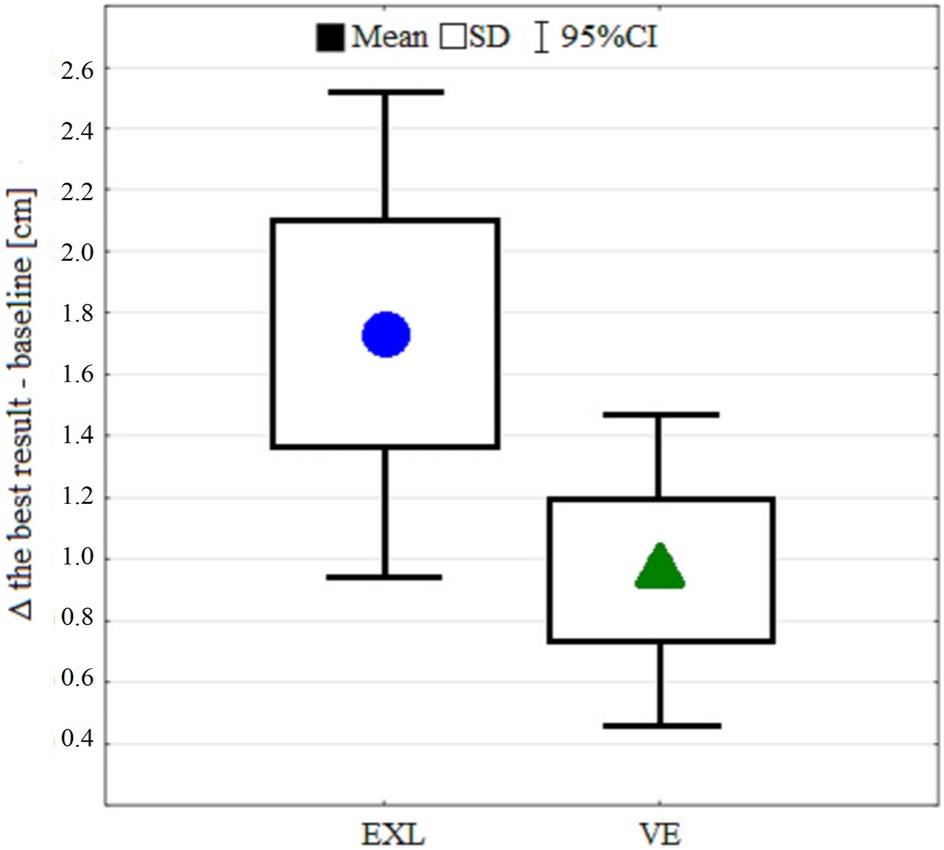
Comparison of absolute improvements between the external load (EXL) and voluntary effort (VE) protocols using the difference (Δ) between the best results and baseline.

To get a clearer picture of the introduced PAPE protocols and to extend the comparison of their effects, an independent sample t-test was conducted for absolute changes (best results and baseline differences) (Fig. 3). However, no significant difference was observed (EXL: 1.9 ± 1.4 cm vs.VE: 1 ± 0.9 cm; t = 2.1; *p =* 0.09) between PAPE protocols, though there was a trend toward more gain in the EXL group (effect size [ES] = 0.76).

## Discussion

This study aimed to assess the effectiveness of isometric PAPE protocols on JH enhancement by examining the effects of protocols using either an EXL or VE. The stated hypothesis was confirmed, with both protocols resulting in significant, though unique, JH improvements. The EXL group peaked faster and declined significantly in the following minutes, whereas the VE group experienced an initial deterioration before peaking nine minutes post-activation. Furthermore, there was a dependency on time to peak performance, with the EXL group peaking in the third minute and the VE group peaking in the ninth minute more frequently. Comparing both protocols’ absolute results (best—baseline) did not show statistically significant differences (though it was close) in JH improvement, with the ES indicating that the EXL tended to be more effective.

Isometric conditioning activity protocols may positively affect acute physical performance enhancement, although there is some ambiguity in the literature^13,15,27,28^. Repeated isometric stimulation of 3–5 s is optimal for performance enhancement^29^. Previous studies have discussed the factors that influence the PAPE effect and have shown that individual characteristics can play a role^30^. In this regard, Seitz and Haff^31^ indicated that PAPE results are impacted by training experience and strength level, with the PAPE effect tending to be more pronounced in stronger individuals following lower volume conditioning activities with shorter recovery intervals. On the other hand, weaker individuals tend to experience increased PAPE effects with longer recovery intervals, multiple-set conditioning activities, and submaximal effort. Considering the groups in the current study had a similar strength level and the disparities in observed jump enhancement between protocols, it can be assumed that the VE protocol was more demanding and longer rest was needed than the EXL protocol. Regardless, the literature comparing the impact of an EXL and VE-based PAPE protocol is limited, especially among females^13,32^.

Using isometric contractions in resistance training effectively develops strength, which translates into acute and long-term dynamic effort improvements^15,33^. Positive PAPE effects were demonstrated in females following dynamic muscle action^12,26^. In contrast, Tsolakis et al.^27^ did not find positive effects of isometric activity in elite female fencers. In our study, both isometric protocols led to improvements but with different patterns. The EXL protocol resulted in faster initial improvement, with a subsequent decrease, while individuals following the VE protocol had worse results initially but showed peak performance in the final minutes.

Using a sub-maximal load in isometric back squats provides optimal stimulation for the muscle and nervous systems while limiting fatigue in males^34,35^. Furthermore, isometric exercises require lower energy expenditure compared to dynamic muscle actions^33,36,37^. However, the positive post-activation potentiation effect is a product of the balance between excitation and fatigue^38^. If deteriorative effects are observed, it can be assumed that such a balance was not achieved. In the current study, the relative EXL used was too high to maintain jump enhancement during testing, as it led to only a brief improvement followed by a decrease. These findings raise doubt over the utility of the proposed protocol in a real-world setting. Therefore, it is necessary to individualize EXLs and assess if lighter loads would be more suitable for females.

Questions arose regarding the VE protocol, although the effectiveness of this approach is supported by the results presented by Spieszny et al.^19^ on CMJ improvements in male team sports players. In our study, improvement occurred a few minutes after an initial deterioration in performance. Volitional maximal effort depends heavily on individual volition, motivation, and personality^21,22^. The study participants were likely fully engaged, resulting in the initial deterioration followed by recovery and subsequent improvement.

Vargas-Molina et al.^18^ demonstrated the positive effects of an EXL protocol in males, though it was important to establish the appropriate volume of effort and load, which should be at least 70–80%-repetition maximum (RM). In our study, peak performance was achieved in the EXL group three to five minutes post-activation, similar to other observations^39,40^. As such, the EXL PAPE protocol improved JH compared to the baseline, although it decreased in the following minutes, perhaps due to fatigue suppressing the PAPE effect^8–10^. Indeed, neural drive improvement in the first minutes could have provided the JH increase in the EXL group, with fatigue occurring in the next minutes leading to a JH decrease^41^. A study by Shafer and Bittman^20^ showed that HIMA with an EXL caused faster fatigue, suggesting that a lower load or volume could avoid fatigue^31^. Liossis et al.^42^ indicated that power output improvement might be achieved in weaker individuals after four minutes of activity with a load of 65%-RM, which also agrees with our study. Regardless, whether the stimulus would be appropriate remains in question. However, Esfromes and Bampouras^43^ showed JH improvement after bodyweight squats, which suggests the possibility of evoking JH enhancement with a lower load.

Several authors have shown JH enhancement after voluntary isometric effort^19,34,44^, with Kalinowski et al.^44^ showing enhanced JH and Spieszny et al.^19^ demonstrating jump improvement among handball payers. Interestingly, peak performance showed wide variability, with some individuals peaking after four minutes and others peaking after eight. These results agree with our observations of peak performance after a similar time. However, the VE decreased JH in the first minutes after the activation protocol, suggesting that participants were maximally engaged in the effort, which may have caused fatigue, although the effort duration was within the suggested limits^45^. These findings raise the question of whether or not the protocol should be shortened or the rest interval increased. In this regard, the data suggests that the rest interval should be extended. Regardless, these conclusions consider the mean results and many studies have shown variability in peak performance^46^. In the current study, ambiguity remains over the influences determining the moment of peak performance.

There are limited data directly comparing isometric protocols using an EXL or VE. The results of the relevant study were not statistically significant, although the EXL group tended to gain more than the VE group. These findings suggest that an EXL could be utilized when a short break is needed (3–5 min) and VE used when a more extended rest is possible. However, it is necessary to pay attention to individuals’ variations.

Caution is required when interpreting the results, as the measurements did not continue beyond the peak performance time of nine minutes, and there is no certainty that the JH would improve in the following minutes. This ambiguity is an obvious limitation of the current study. The lack of a force platform for voluntary isometric protocol assessment of individual engagement and having no body temperature measurement also limited the study. We did not directly measure the power and force involved during the jump, which would provide more insight into the results obtained. Nonetheless, the existing literature comparing an EXL to VE in females is lacking, and this work provides some new insights.

Future studies need to clarify if applying a lower EXL would provide a similar stimulus for JH improvement without deteriorative effects on peak performance. Also, observation time should be prolonged in the VE protocol due to late peak performance occurrence, which may provide more insight. In addition, there is a need to directly measure the force and power involved during jumping to extend the observations.

## Conclusions

Both PAPE protocols improved JH, although they also had some disadvantages. The protocol with a relative EXL enhanced JH after three minutes, followed by a decrease. In contrast, the protocol based on VE led to a deteriorative effect on JH in the first minutes and a peak after nine minutes. As such, both protocols offer positive and negative aspects and limited utility in some settings. Therefore, caution is needed when utilizing the protocols, and individual responses to the proposed protocols should be sought. Our findings have implications for practitioners using isometric PAPE protocols with an external submaximal load or VE among females. Both protocols can provide enhancement of dynamic exercises, such as vertical jump, and be used in direct preparation for dynamic efforts. However, their usefulness is limited due to possible deteriorative effects, and caution is warranted.

When there is a requirement for rapid achievement of physical performance enhancement, a protocol with a submaximal external (70%-RM) load and a shorter break is suggested, and when prolonged rest is possible, a VE can be performed. However, verifying the effects in a training setting, establishing individual responses to the chosen protocol, and determining peak performance time are suggested due to potential individual variability.

## Material and methods

### Experimental design

The first (familiarization) session familiarized participants with the CMJ demands, verified their 1-RM in the back squat, introduced an appropriate conditioning activity, and divided them into one of three groups, including EXL, VE, and control (CON). At the second (experimental) session, participants completed a standardized warm-up, then performed CMJ baseline measures, and the PAPE protocols were introduced according to group adherence. After that, participants performed CMJs at fixed intervals.

### Participants

The Ethics Committee of the Wroclaw University of Sport Health Sciences approved the study (06/2023), which followed the guidelines of the Declaration of Helsinki. All participants volunteered, were familiarized with the study procedure and associated experimental risks, provided written informed consent to participate, and could abandon the investigation at any moment. They were also required to complete a medical questionnaire regarding their health status in terms of any musculoskeletal injury history and cardiovascular, neurological, metabolic, and other issues.

Participants were asked to refrain from exhaustive physical effort, alcohol consumption, and intake of ergogenic compounds such as caffeine for 48 h before the study. However, typical sleeping, eating, and drinking habits were to be maintained. The follow-up stage included 53 recreationally resistance trained females, reduced to 46 after applying inclusion–exclusion criteria. While participation in other recreational activities was permitted, competitive sports involvement was strictly prohibited among the participants. The inclusion criteria were age 19–23, lack of musculoskeletal injury eight weeks before the study, no other medical contradiction, at least six years of experience in strength training, and the ability to complete back squats at 100–130% of body weight. Not meeting at least one of the criteria resulted in disqualification from the study.

Participants were randomly divided into three groups during the familiarization session using the www.randomizer.org tool. Simple random sampling without replacement was used. The sampling frame included a list of participants alphabetically ordered and coded by numbers 1–46. Five individuals abandoned the experiment for reasons not associated with the study. Therefore, the final groups included EXL (n = 15), VE (n = 14), and CON (n = 12).

### Measurements

#### Body morphology

Body heights were measured with an anthropometer (GPM Anthropological Instruments, DKSH Ltd., Zürich, Switzerland) to the nearest 0.1 cm. The measurements were carried out in accordance with the anthropometric measurement guidelines established by the International Society for the Advancement of Kinanthropometry (ISAK), ensuring adherence to standardized procedures. Body height was measured without shoes and socks, with individuals standing with their arms hanging naturally along the torso, their head positioned in the Frankfurt plane, and with heels, buttocks, back, and back of the head touching the anthropometer. The individual being measured took a deep breath during measurement^47^.

An InBody230 device (InBody Co., Ltd., Cerritos, CA, USA) assessed body mass to the nearest 0.1 kg. The device has confirmed reliability^48^. During the measurement process, strict adherence to the standardized conditions for bio-impedance (BIA) measurement was ensured, as outlined by Kyle et al.^49^. The measurements were taken between 7 and 11 a.m. Participants were instructed to refrain from engaging in physical activity and consuming food or beverages for a minimum of eight hours before the measurements and to avoid emptying their bladders immediately before the assessment. Participants positioned themselves on the device’s platform without footwear, ensuring that the soles of their feet made contact with the electrodes. Additionally, they held onto the unit’s handles with their thumb and fingers to establish direct contact with the electrodes. Throughout the assessment, participants maintained a stationary posture, with their elbows fully extended and their shoulder joints slightly abducted at an angle of approximately 30°. Body Mass Index (BMI) was then calculated based on the formula: BMI = kg/m^2^.

### The one maximum repetition assessment (1-RM)

The load-velocity association established the back squat 1-RM using the Vitruve VBT device (Vitruve, SPEED4LIFTS S.L., Madrid, Spain)^50,51^, for which reliability was confirmed^52^. Participants’ general warm-up included a five-minute run at 6 km/h, dynamic joint mobilization, and two rounds of ten repetitions of body squats, lunges, and hip thrusts, with a 60-s break between each round. The warm-up involved a maximal deep-back squat with an empty bar to establish the maximal individual depth while maintaining proper technique and a safe body position. Every repetition of the back squat was demanded and controlled by parallels set at the corresponding height, and the investigator was present. The first warm-up set involved 12–15 repetitions with an empty bar and was followed by a second set of 10–12 repetitions at 50%-RM (mean velocity of 1.0–1.2 m/s). Participants then performed two to three sets of five repetitions at 60–80%-RM (mean velocity of 0.5–0.75 m/s). After achieving a mean velocity of 0.5 m/s, a progressive incremental test commenced. The test used an initial load of 80%-RM for three reps (0.5 m/s mean velocity), with 5–10% load (kg) increases in the following sets. When the mean velocity decreased below 0.5 m/s, each set included two repetitions, and when it fell below 0.25 m/s, sets included one repetition. The test proceeded for a maximum of five sets or until failure occurred, with a three to five-minute break between sets.

### Countermovement jump

The countermovement jump (CMJ) was performed according to principles presented by Comfort et al.^53^ and assessed using the OptoJump device (Microgate, Bolzano, Italy). The reliability of chosen device is confirmed^54^. Baseline JH was measured before the PAPE protocol and then at three, five, seven, and nine minutes after the conditioning activity. The participants were instructed to assume an upright position with equal weight distribution on both feet. Hands were placed on the hips and remained in that position throughout the whole test. Individuals then performed a squat, bending the knees to approximately 90 degrees, followed immediately by a vertical jump with the aim of achieving maximum height. Participants were encouraged to perform the countermovement as quickly as possible and maintain the same position in every trial. Individuals adopt their preferred depth of countermovement what is considered the most effective approach for ensuring consistency and reliability in individuals’ performance what showed Petronijevic et al.^55^. The landing required both feet to land simultaneously. Participants were instructed to maintain the same depth in each trial, ensuring symmetrical take-offs from both lower limbs and controlled landing to amortize the impact. During the flight phase, flexion of the lower limbs (knee and/or hip joints) was not permitted^54^.

### Conditioning activity

EXL, VE, and CON groups performed the same standardized warm-up, including a five-minute treadmill jog at 6 km/h, joint mobilization, and two rounds of ten repetitions of body squats, lunges, and hip thrusts, with a 60-s break between each round. Additionally, participants completed three CMJs, with a 30-s rest between trials. The baseline CMJ height was then recorded after a three-minute rest. After a further 60 s, the conditioning protocols were introduced. The EXL group performed a conditioning activity based on isometric effort, which included three four-second sets of the full-back squat at 70%-RM, with 60-s breaks. The VE group protocol involved three five-second sets of maximal isometric contraction evoked by pushing as strongly as possible on an immovable bar, with a one-minute rest interval. The CON group performed a five-minute treadmill run at 6 km/h to maintain preparedness for the trial. CMJ trials followed the protocols for each group.

### Statistics

Power analysis determined the sample size using GPower version 3.1.9.6 software (Heinrich Heine University, Düsseldorf, Germany). Assuming 80% power, a minimum ES of 0.20, and α = 0.05, the minimum required sample size for repeated measures ANOVA of within and between group interactions was 36 subjects^56,57^. The chi-squared test assessed participant distribution between groups (15 vs. 14 vs. 12).

Statistical analysis employed Statistica 13.0 software (StatSoft Poland, Krakow, Poland), with results expressed as mean ± standard deviation (SD) and 95% confidence intervals (95% CIs). The Shapiro–Wilk test examined the data distribution, while Levene’s test confirmed the homogeneity of variance. A one-way ANOVA determined differences in basic parameters between groups, and a two-factor repeated-measures ANOVA compared differences in JH and time between groups, providing η^2^ values. If differences became apparent, Duncan’s post hoc was performed, while the chi-squared test detected the significance of the time to peak performance. The final analysis compared the relative change in JH from baseline to the best result using Student’s t-test for independent samples. Cohen’s d values for ES were calculated and interpreted as small (ES ≤ 0.2), medium (ES ≤ 0.5–0.79), or large (ES ≥ 0.8)^58^. The significance level was set at *p <* 0.05.

## Data Availability

The datasets generated during and/or analyzed during the current study are available from the corresponding author on reasonable request.
